# An Electrophilic Deguelin Analogue Inhibits STAT3 Signaling in H-*Ras*-Transformed Human Mammary Epithelial Cells: The Cysteine 259 Residue as a Potential Target

**DOI:** 10.3390/biomedicines8100407

**Published:** 2020-10-12

**Authors:** Sung-Jun Hong, Jin-Tae Kim, Su-Jung Kim, Nam-Chul Cho, Kyeojin Kim, Seungbeom Lee, Young-Ger Suh, Kyung-Cho Cho, Kwang Pyo Kim, Young-Joon Surh

**Affiliations:** 1 Department of Molecular Medicine and Biopharmaceutical Sciences, Graduate School of Convergence Sciences and Technology, Seoul National University, Seoul 08826, Korea; serlephace@naver.com; 2 Tumor Microenvironment Global Core Research Center, College of Pharmacy, Seoul National University, Seoul 08826, Korea; yorybogo@naver.com (J.-T.K.); nynna79@snu.ac.kr (S.-J.K.); kyeojin01@gmail.com (K.K.); lastchaos21c@snu.ac.kr (S.L.); ygsuh@snu.ac.kr (Y.-G.S.); 3 Korea Chemical Bank, Korea Research Institute of Chemical Technology, Daejeon 34114, Korea; cord80@naver.com; 4 Department of Applied Chemistry, Institute of Natural Science, Global Center for Pharmaceutical Ingredient Materials, Kyung Hee University, Youngin 17104, Korea; chokc@snu.ac.kr (K.-C.C.); kimkp@khu.ac.kr (K.P.K.); 5 Department of Biomedical Science and Technology, Kyung Hee Medical Science Research Institute, Kyung Hee University, Seoul 02447, Korea; 6 Cancer Research Institute, Seoul National University, Seoul 03080, Korea

**Keywords:** STAT3, deguelin, SH48, α,β-unsaturated carbonyl group, MCF10A-*ras* cells, autophagy

## Abstract

Signal transducer and activator of transcription 3 (STAT3) is a point of convergence for numerous oncogenic signals that are often constitutively activated in many cancerous or transformed cells and some stromal cells in the tumor microenvironment. Persistent STAT3 activation in malignant cells stimulates proliferation, survival, angiogenesis, invasion, and tumor-promoting inflammation. STAT3 undergoes activation through phosphorylation on tyrosine 705, which facilitates its dimerization. Dimeric STAT3 translocates to the nucleus, where it regulates the transcription of genes involved in cell proliferation, survival, etc. In the present study, a synthetic deguelin analogue SH48, discovered by virtual screening, inhibited the phosphorylation, nuclear translocation, and transcriptional activity of STAT3 in H-*ras* transformed human mammary epithelial MCF-10A cells (MCF10A-*ras*). We speculated that SH48 bearing an α,β-unsaturated carbonyl group could interact with a thiol residue of STAT3, thereby inactivating this transcription factor. Non-electrophilic analogues of SH48 failed to inhibit STAT3 activation, lending support to the above supposition. By utilizing a biotinylated SH48, we were able to demonstrate the complex formation between SH48 and STAT3. SH48 treatment to MCF10A-*ras* cells induced autophagy, which was verified by staining with a fluorescent acidotropic probe, LysoTracker Red, as well as upregulating the expression of LC3II and p62. In conclusion, the electrophilic analogue of deguelin interacts with STAT3 and inhibits its activation in MCF10A-*ras* cells, which may account for its induction of autophagic death.

## 1. Introduction

Growing attention has been focused on the identification of natural products with chemopreventive and chemotherapeutic potential [1,2]. Deguelin is a natural rotenoid that is isolated from several medicinal plants, including *Derris trifoliata Lour* (Leguminosae), *Mundulea sericea* (Leguminosae), and *Tephrosia vogelii Hook.f* (Leguminosae) [3]. Deguelin inhibits the proliferation and growth of diverse malignant cells, including head and neck [4,5], lung [6], esophageal [7], gastric [8]. colon [9], pancreatic [10,11], prostate [12], and breast [13,14] cancer cells by targeting multiple signaling molecules. Although deguelin has promising chemopreventive and chemotherapeutic potential [15,16], undesired side effects and toxicity limit its clinical application [17,18]. To overcome such limitations, a series of synthetic analogues of deguelin have been synthesized to evaluate their anticancer activities [19].

Signal transducer and activator of transcription (STAT) family proteins play important roles in cell growth, survival, and immunity [20,21,22]. Among these, STAT3 is a transcription factor that is involved in tumor development and progression as well as inflammation [22,23,24,25]. STAT3 is constitutively activated in several cancer cells [23,24,25,26]. The aberrantly activated STAT3 is believed to contribute to malignant transformation at several levels, including uncontrolled proliferation through activation of several cell-cycle regulators, (e.g., Cyclin D1 and c-Myc) as well as the evasion of apoptosis by inducing the expression of anti-apoptotic proteins (e.g., Bcl-xL, Bcl-2, Mcl-1, and Survivin) [24,25,26]. STAT3 also mediates the expression of proteins involved in other hallmarks of cancer, such as invasion/metastasis [27] and angiogenesis [28]. Therefore, STAT3 is considered to be an important target for the development of cancer therapeutic agents [23,25,26,29,30,31,32,33]. The suppression of a STAT3 signal pathway by deguelin has been observed in adult T cells transformed by human T-cell leukemia virus [34].

Autophagy is a self-eating mechanism [35]. In autophagy, a phagophore engulfs intracellular organelles or aberrant proteins and then makes autophagosome with microtubule-associated protein 1A/-light chain 3II (LC3II) and p62 [36,37]. Autophagosome fused with lysosomes becomes autolysosome [36,37]. Autolysosome is eventually degraded, and it supplies nutrition to the surrounding environment. It has been reported that STAT3 represses autophagy in human cells, while the inhibition of STAT3 potently stimulates the autophagic flux and thereby affects the fate of cells [38,39]. In a previous study, a series of deguelin analogues were synthesized to develop novel STAT3 inhibitors [40]. Here, we report that an electrophilic derivative of deguelin inactivates STAT3 through complex formation and induces autophagy in human breast epithelial cells transformed by H-*ras* oncogene (MCF10A-*ras*).

## 2. Experimental Section

### 2.1. Materials

SH48 (M.W. 380.43) and its derivatives were synthesized as described previously [40]. Dulbecco’s modified Eagle’s medium (DMEM)/Ham’s nutrient mixture F-12 (1:1), Rosewell Park Memorial Institute (RPMI) 1640 medium, Dulbecco’s modified Eagle’s medium (DMEM), fetal bovine serum (FBS), and horse serum were obtained from Gibco BRL (Grand Island, NY, USA). Cholera toxin, hydrocortisone, insulin, human epidermal growth factor (h-EGF), and 3-(4,5-dimethylthiazol-2-yl)-2,5-diphenyltetrazolium bromide (MTT) were purchased from Sigma-Aldrich Co. (St. Louis, MO, USA). p-STAT3-TA-Luc plasmid was purchased from BD Biosciences Clontech (San Jose, CA, USA). Antibodies against p-STAT3, STAT3, LC3II, p62, Bcl-xL, human influenza hemagglutinin (HA)-tag, and Myc-tag were obtained from Cell Signaling Technology (Beverly, MA, USA). Agarose immunoprecipitation reagents were products of Santa Cruz Biotechnolgies Co. (Santa Cruz, CA, USA). Secondary antibodies, STAT3 short interfering RNA (siRNA), negative control siRNA, and lipofectamin RNAi-MAX reagent were purchased from Invitrogen Life Technologies, Inc. (Carlsbad, CA, USA). All other chemicals used were of analytical or the highest purity grade available.

### 2.2. Cell Culture

MCF10A-*ras* cells were grown in DMEM/F-12 medium supplemented with 5% heat-inactivated horse serum, insulin (10 μg/mL), cholera toxin (100 ng/mL), hydrocortisone (0.5 μg/mL), h-EGF (20 ng/mL), L-glutamine (2 mmol/L), and penicillin/streptomycin (100 units/mL). Human prostate cancer PC3 cells were cultured in RPMI medium supplemented with 10% (*v*/*v*) heat-inactivated FBS, penicillin (100 U/mL), and streptomycin (100 g/mL). HeLa/STAT3-luc cells were maintained in DMEM supplemented with 10% FBS and an 100 ng/mL penicillin/streptomycin/fungizone mixture. The cells were grown at 37 °C in a humidified air/CO_2_ (19:1) atmosphere. The cells were plated at an appropriate density according to each experimental scale.

### 2.3. MTT Reduction Assay

The cell viability was determined by the MTT reduction assay. MCF10A-*ras* cells plated at a density of 2.5 × 10^4^ cells/200 μL in 48-well plates were treated with SH48 and its analogues. After incubation for 24 h, cells were treated with the MTT solution (final concentration, 1 mg/mL) for an additional 2 h. The dark blue formazan crystals formed in intact cells were dissolved with dimethyl sulfoxide (DMSO), and the absorbance at 570 nm was read using a microplate reader.

### 2.4. Transient Transfection and the Luciferase Reporter Assay

HeLa/STAT3-luc cells were seeded at a density of 2 × 10^5^ per well in a six-well dish and grown to 60% to 80% confluence in the complete growth medium. Cells were pretreated with test compounds for 24 h and stimulated with 10 ng/mL oncostatin M (OSM) for 5 h. The cells were then washed with phosphate-buffered saline (PBS) and lysed in 1x reporter lysis buffer (Promega). The lysed cell extract (20 µL) was mixed with 100 µL of the luciferase assay reagent, and the luciferase activity was determined using a luminometer (AutoLumat LB 953, EG&G Berthold). The β-galactosidase activity was measured to normalize the luciferase activity.

### 2.5. siRNA Knockdown of STAT3.

The human STAT3 siRNA duplex and negative control siRNA were purchased from KDR Biotech Co., Ltd. (Seoul, South Korea). The sequences of each siRNA were as follows: STAT3 siRNA-1 (sense, 5′UGUUCUCUGAGACCCAUGAdTdT-3′; antisense, 5′-UCAUGGGUCUCAGAGAACAdTdT-3′).

### 2.6. Western Blot Analysis

MCF10A-*ras* cells were lysed in lysis buffer (250 mmol/L sucrose, 50 mmol/L Tris-HCl (pH 8.0), 25 mmol/L KCl, 5 mmol/L MgCl_2_, 1 mmol/L EDTA, 2 mmol/L NaF, 2 mmol/L sodium orthovanadate, and 1 mmol/L phenylmethylsulfonylfluoride (PMSF)) for 15 min on ice followed by centrifugation at 13,000× *g* for 15 min. The BCA reagent (Pierce Biotechnology; Rockfold, IL, USA) was used to measure the protein concentrations. Protein (30 μg) was separated on 8% sodium dodecyl sulfate polyacrylamide gel electrophoresis (SDS-PAGE) gel and transferred to the polyvinylidene fluoride (PVDF) membrane (Gelman Laboratory; Ann Arbor, MI, USA). The blots were blocked with 5% nonfat dry milk PBS containing 0.1% (*v*/*v*) Tween-20 (PBST) for 1 h at room temperature. The membranes were incubated for 2 h at room temperature with an 1:1000 dilution of antibodies for p-STAT3, STAT3, poly (ADP-ribose) polymerase (PARP), Sequestosome 1 (SQSTM1)/p62, Bcl-_X_L, and LC3B (Cell Signaling Technology; Beverly, MA, USA). Actin was used to ensure equal lane loading. The blots were rinsed three times with PBST for 10 min each. Washed blots were treated with an 1:5000 dilution of horseradish peroxidase (HRP) conjugated-secondary antibody (Pierce Biotechnology; Rockford, IL, USA) for 1 h and washed again three times with PBST. The transferred proteins were visualized with an enhanced chemiluminescence detection kit (Amersham Pharmacia Biotech; Buckinghamshire, UK).

### 2.7. Immunoprecipitation

After treatment with biotinylated SH48 for 24 h, MCF10A-*ras* cells were lysed in 250 mmol/L sucrose, 50 mmol/L Tris-HCl (pH 8.0), 25 mmol/L KCl, 5 mmol/L MgCl_2_, 1 mmol/L EDTA, 2 mmol/L NaF, 2mmol/L sodium orthovanadate, and 1 mmol/L PMSF. Total protein (500 μg) was immunoprecipitated by shaking with STAT3 primary antibody at 4 °C for 12 h followed by the addition of protein A/G-agarose bead suspension (25% slurry, 20 mL) and additional shaking for 2 h at 4 °C. After centrifugation at 10,000 rpm for 1 min, immunoprecipitated beads were collected by discarding the supernatant and washed with cell lysis buffer. The immunoprecipitate was then resuspended in 8 μL of 6 × SDS electrophoresis sample buffer and boiled for 5 min. Supernatant (48 μL) from each sample was collected by centrifugation and loaded on SDS-polyacrylamide gel. The incorporation of biotinylated SH48 into immunoprecipitated proteins was visualized by use of Amersham streptavidin–HRP conjugate.

### 2.8. Immunocytochemistry

Transfected PC3 cells were plated on the chamber slide at a density of 5 × 10^4^ cells/mL and treated with SH48 or DMSO. Cells were fixed with 95% methanol/5% acetic acid at −20 °C for 5 min, washed with PBS twice, treated with 0.2% Triton X-100 in PBS for 5 min, and washed with PBST and then with PBS. Samples were incubated with blocking agent (0.1% Tween-20 in PBS containing 5% bovine serum albumin (BSA)) at room temperature for 1 h, washed with PBS, and then incubated with a diluted (1:100) primary antibody for LC3II (Cell Signaling Technology, #2775) overnight at 4 °C. After washing with PBS, samples were incubated with a diluted (1:1000) tetramethylrhodamine (TRITC)-conjugated anti-mouse or fluorescein isothiocyanate (FITC)-conjugated anti-rabbit IgG secondary antibody in PBST containing 1% BSA at room temperature for 1 h. Samples were washed with 0.1% PBST containing 1% BSA and examined under a fluorescent microscope.

### 2.9. Measurement of the Autophagy

Following treatment, cells were rinsed with PBS and were incubated with 100 nM LysoTracker Red DND-99 (Thermo Fisher Scientific, Waltham, MA, USA.). After 30 min incubation at 37 °C, the cells were visualized with fluorescence microscopy.

### 2.10. Molecular Modeling

Binding mode analysis of SH48 was carried out using a covalent docking module implemented in Maestro program v9.5 (Schrödinger LLC; New York, NY, USA). The crystal structure of STAT3 protein (PDB code: 1BG1) was downloaded from PDB bank. The STAT3 structure was prepared with neutralization and energy minimization using ProteinPrep Wizard. SH48 was prepared with protonation at pH 7.4 and energy minimization using LigPrep module. In a covalent docking module, the reaction type of the ligand was set to Micheal addition with cysteine 259 of STAT3 protein. A grid box was generated with residues within 5.0 Å of cysteine 259. The 10 initial outputs of the covalent binding pose were energetically minimized with residues within 3 Å of SH48, and their binding energy was calculated by Prime MM-GBSA module. The covalent binding pose of SH48 was selected with low PrimeΔG_bind_ for binding mode analysis.

### 2.11. Statistical Analysis

Data were expressed as means ± SD of at least three independent experiments, and statistical analysis for single comparison was performed using the Student’s *t-*test. The statistical significance was considered when *p* < 0.05.

## 3. Results

### 3.1. Comparative Effect of Deguelin Analogues on STAT3 Transcriptional Activity

**Figure 1:**
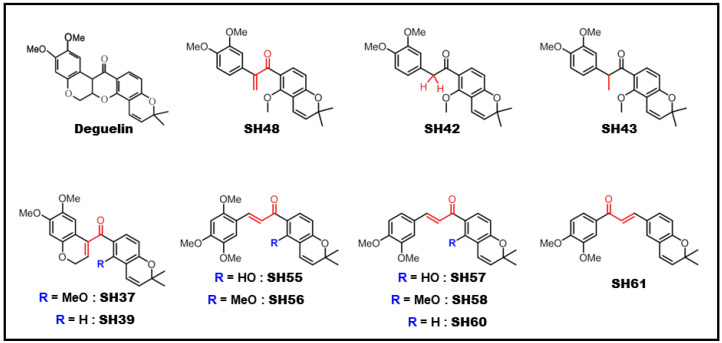
Chemical structures of synthetic deguelin and its synthetic derivatives tested in this study.

**Figure 2:**
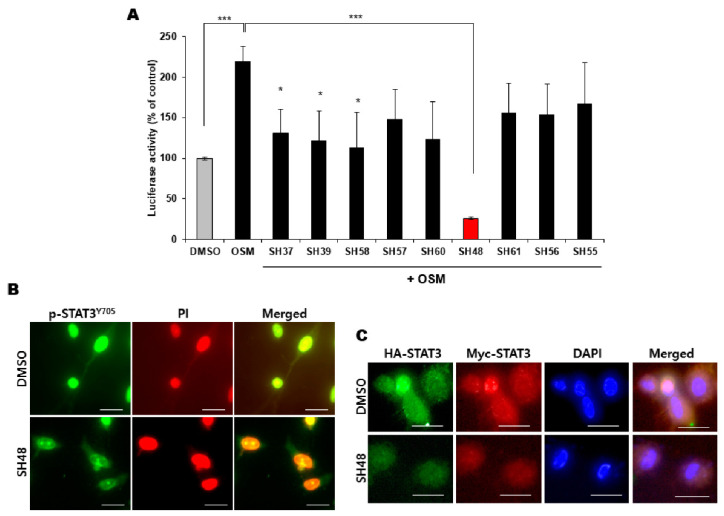
Effects of deguelin analogues on transcriptional activity, nuclear localization and dimerization of signal transducer and activator of transcription 3 (STAT3). (**A**) HeLa/STAT3-luc cells were pretreated with dimethyl sulfoxide (DMSO) or each of analogues (10 μM) for 24 h, stimulated with 10 ng/mL of oncostatin M (OSM) for another 5 h, and then assayed for the luciferase reporter gene activity as described in Section 2. * *p* < 0.05; *** *p* < 0.001. (**B**) Immunocytochemical analysis was performed using p-STAT3^Y705^ antibody after the treatment of MCF10A-*ras* cells with 10 μM SH48 for 24 h. Cells were stained with PI and visualized by confocal microscopy. Scale bar, 50 μm. (**C**) Immunocytochemical analysis was performed using HA-tag and Myc-tag antibodies. PC3 cells were treated with 10 μM SH48 for 24 h. Cells were stained with DAPI and analyzed by confocal microscopy. Scale bar, 50 μm.

Several analogues with a modified deguelin skeletal structure (Figure 1) were prepared. To find out which of these compounds was most effective for suppressing STAT3 signaling, their effects on STAT3 transcriptional activity were assessed by the luciferase reporter gene assay. The HeLa/STAT3-luc cell line was pretreated with each compound for 24 h and stimulated with OSM (10 ng/mL) for an additional 5 h. As shown in Figure 2A, SH48 was found to be the most potent inhibitor of STAT3 transcriptional activity.

STAT3 phosphorylation at the tyrosine 705 residue facilitates STAT3 dimer formation, leading to nuclear translocation and binding to a specific DNA site to regulate target gene transcription [5]. Therefore, the inhibition of the homodimerization of STAT3 has been considered as a rational strategy in the management of cancer [41]. STAT3 phosphorylated at the tyrosine 705 (p-STAT3^Y705^) stained with FITC-conjugated anti-rabbit IgG antibody was visualized as green fluorescence. The result of immunocytochemistry showed that SH48 treatment reduced the levels of p-STAT3^Y705^ and its nuclear translocation at the same concentration which inhibited STAT3 transcriptional activity (Figure 2B). In another experiment, STAT3 was tagged with HA and Myc. HA-tagged STAT3 was detected by green fluorescence, and Myc-tagged STAT3 was indicated by red fluorescence. The nuclear marker DAPI was shown in blue fluorescence. In SH48-treated cells, a bright blue color of the nucleus was seen, while green or red colors were less intense (Figure 2C). These findings suggest that SH48 inhibits STAT3 dimerization and translocation into the nucleus.

### 3.2. The α,β-Unsaturated Carbonyl Moiety of SH48 Is Essential for the Inhibition of STAT3 Phosphorylation

**Figure 3:**
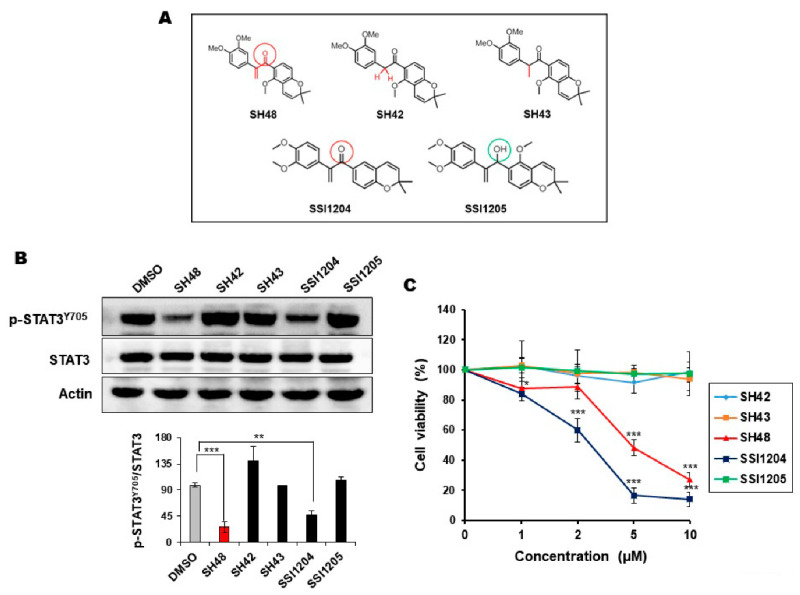
Effects of selected deguelin analogues on the phosphorylation of STAT3 and cell viability. (**A**) Chemical structures of SH48 and SH1203 and their non-electrophilic analogues. Red and green circles denote electrophilic carbonyl and non-electrophilic alcoholic functions, respectively. (**B**) MCF10A-*ras* cells were treated with 5 μM each of SH48 SH42, SH43, SSI-1204, and SSI-1205 for 24 h, and the expression of P-STAT3 and STAT3 was measured by Western blot analysis. Actin was measured for the confirmation of the equal amount of loaded protein. ** *p* < 0.01; *** *p* < 0.001. (**C**) MCF10A-*ras* cells were treated with indicated concentrations of SH42, SH43, SH48, SSI1204, and SSI1205 for 24 h. The cell viability was measured by the 3-(4,5-dimethylthiazol-2-yl)-2,5-diphenyltetrazolium bromide (MTT) assay as described in Section 2. * *p* < 0.05; *** *p* < 0.001.

It is known that the α,β-unsaturated carbonyl group is required for the inhibition of STAT3 phosphorylation by some chalcone derivatives [42]. MCF10A-*ras* cells were treated with a 5 μM concentration of SH48, SH42, SH43, SSI1204, or SSI1205 for 24 h. SH42 has a substitution in a double bond with hydrogen in comparison with SH48. SH43 has a single bond. As with SH48, SSI1204 has an α,β-unsaturated carbonyl moiety (Figure 3A). SSI1205 has a substitution in the carbonyl group with a hydroxyl group and some addition in a benzene ring (Figure 3A). Then, the protein expression of p-STAT3 as well as STAT3 was measured by Western blot analysis. As shown in Figure 3B, STAT3 phosphorylation was significantly decreased by treatment with SH48 and SSI1204 harboring an electrophilic α,β-unsaturated carbonyl moiety. However, total STAT3 expression was not affected (Figure 3B). In parallel with the inhibition of STAT3 phosphorylation, both SH48 and SSI1204 reduced the viability of MCF10A-*ras* cells, whereas the other analogues without an electrophilic moiety were not cytotoxic (Figure 3C).

### 3.3. SH48 Forms a Complex with STAT3

**Figure 4:**
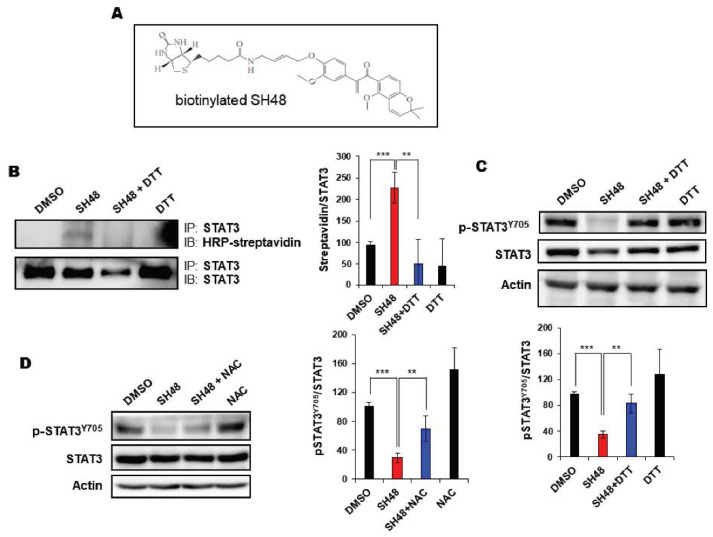
Effects of thiol reducing agents on interaction of SH48 with STAT3 and phosphorylation of STAT3. (**A**) Structure of biotinylated SH48. (**B**) MCF10A-*ras* cells were incubated with 30 for 24 h. MCF10A-*ras* cells were pretreated with 0.5 mM dithiothreitol (DTT) for 1 h and treated with the biotin conjugate of SH48 (30 μM) for an additional 24 h. The complex formed between the biotin-conjugate of SH48 to STAT3 was detected by immunoblot analysis using horseradish peroxidase (HRP)–streptavidin. ** *p* < 0.01; *** *p* < 0.001. (**C**,**D**) MCF10A-*ras* cells were pretreated with 0.5 mM DTT (**C**) or 5 mM N-acetylcysteine (NAC) (**D**) for 1 h, followed by exposure to SH48 (10 μM) for additional 24 h. The interaction between STAT3 and SH48 was assessed as described above. ** *p* < 0.01; *** *p* < 0.001.

To demonstrate the direct interaction between STAT3 and SH48, a biotinylated SH48 was used (Figure 4A). The binding of biotin-conjugated SH48 to STAT3 was detected with HRP–streptavidin (Figure 4B). Treatment with the thiol-reducing agent dithiothreitol (DTT) abolished the interaction between SH48 and STAT3 (Figure 4B), suggesting the possible modification of STAT3 cysteine thiol by SH48. Likewise, the SH48-induced suppression of STAT3 phosphorylation was attenuated by DTT (Figure 4C) and also by another thiol-reducing agent and N-acetylcysteine (NAC) (Figure 4D). This observation suggests a possible interaction of SH48 with a cysteine residue present in STAT3.

### 3.4. Cysteine 259 of STAT3 Is a Putative Binding Site of SH48

**Figure 5:**
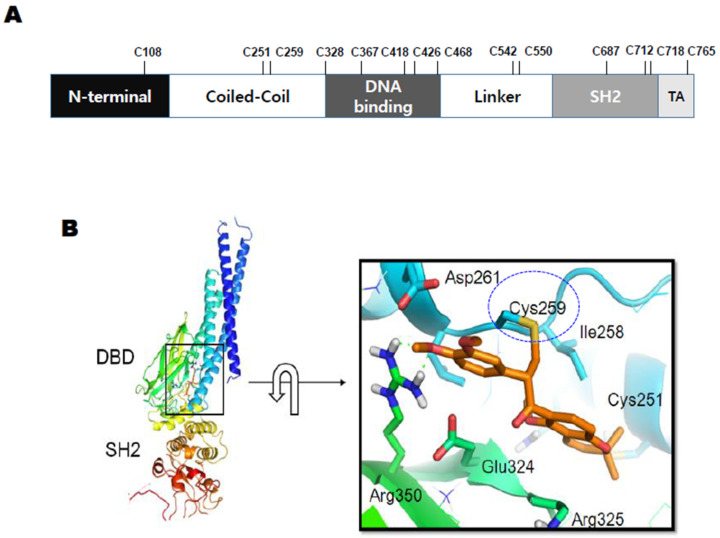
Putative interaction between the cysteine 259 residue of STAT3 and SH48 determined by computer modeling. (**A**) Cysteine residues present in human STAT3. SH2, Src Homology 2 domain; TA, transactivation domain. (**B**) The covalent binding pose of the α,β-unsaturated carbonyl group of SH48 to Cys259 of STAT3. DBD, DNA-binding domain; SH2, Src Homology 2 domain.

**Figure 6:**
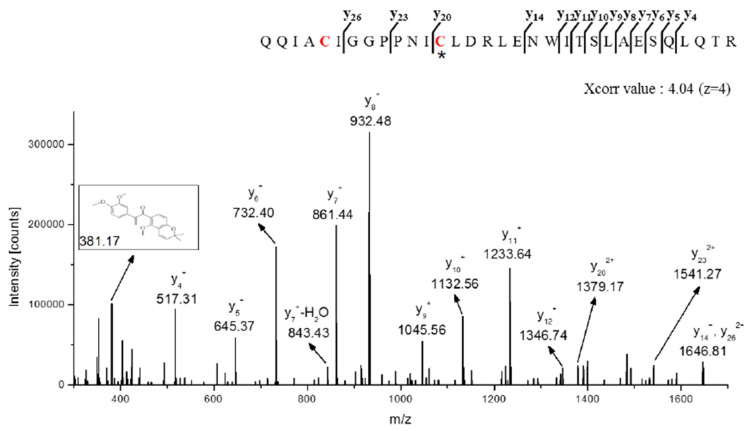
Mass spectrometric analysis of STAT3 modified by SH48. Mass spectrum of the STAT3 peptide (QQIACIGGPPNI**C**^259^LDRLENWITSLAESQLQTR) with SH48 binding. The annotated tandem mass spectroscopy (MS/MS) spectrum illustrating the binding of SH48 to STAT3 protein at cysteine 259 residues was identified by liquid chromatography–tandem mass spectroscopy (LC-MS/MS). Each symbol present with b and y ions on the spectrum represents the SH48 fragment ion bound to Cys259. *C denotes Cys259 modified by SH48.

There are 14 cysteine residues present in human STAT3 (Figure 5A). A computer structure modeling program showed the cysteine 259 residue of STAT3 to be a putative binding site of SH48 (Figure 5B). An interaction between SH48 and Cys259 of STAT3 was anticipated based on the fact that this amino acid is located in the short loop between helices α2 and α3 of the amino-terminal four-helix bundle of STAT3, which lies against the core β-barrel of the monomer. Therefore, Cys259 is exposed in the surface of the tertiary structure of STAT3 (Figure 5B) and considered to be prone to disulfide bridge formation upon oxidative stress [43]. The mass spectrometric analysis also indicated an interaction between SH48 and Cys259 of STAT3 (Figure 6).

### 3.5. SH48 Induces Autophagy

**Figure 7:**
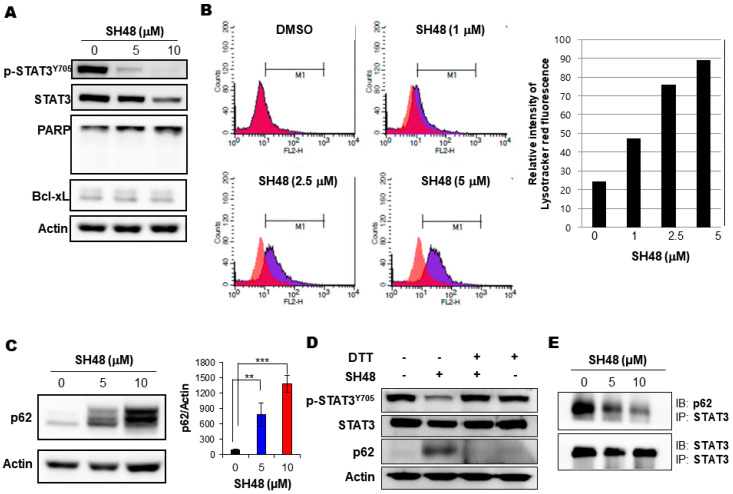
Induction of autophagy by SH48. (**A**) MCF10A-*ras* cells were treated with SH48 for 24 h. Lysates were immunoblotted with the indicated antibodies. (**B**) MCF10A-*ras* cells were treated with indicated concentrations of SH48 for 24 h and then incubated with 100 nM LysoTracker Red for 30 min. The cells undergoing autophagic death were identified by FACS analysis. (**C**) SH48-induced expression of autophagy marker proteins, LC3II and p62 by Western blot analysis. ** *p* < 0.01; *** *p* < 0.001. (**D**) Effects of the thiol-reducing agent DTT on SH48-induced STAT3 phosphorylation and p62 expression. MCF10A-*ras* cells pretreated with 0.5 mM DTT for 1 h, followed by 10 μM SH48 for 24 h. (**E**) Direct interaction between p62 and its inhibition by SH48. MCF10A-*ras* cells were treated with indicated concentrations of SH48 for 24 h. Protein lysates were immunoprecipitated with STAT3 antibody followed by Western blot analysis with anti-p62 antibody.

The suppression of STAT3 has been shown to induce apoptosis [44]. We determined whether a cytotoxic dose of SH48 could induce the apoptosis of MCF10A-*ras* cells overexpressing STAT3; however, we were unable to detect hallmarks of apoptosis, such as the cleavage of PARP and reduction in the levels of the anti-apoptotic protein Bcl-xL (Figure 7A). For this reason, SH48-induced cell death is unlikely to be mediated by apoptosis.

Autophagy is a self-eating mechanism whereby cells digest themselves during starvation. During autophagy, the intracellular phagophore engulfs misfolded proteins of cellular organelles and then forms autophagosome. Autophagic vesicles and their contents are destroyed by the fusion of lysosomes. The role of autophagy in cancer is complex. Autophagic vesicles reflect the existence of type-II cell death, or instead an adaptive response to maintain continual cell survival under stress condition [45]. It is known that STAT3 can interfere with autophagic pathways, and that the suppression of STAT3 induces autophagy [38,39]. SH48-induced autophagic cell death was assessed by flowcytometric analysis of autolysosome formation using a fluorescent acidotropic probe LysoTracker Red (Figure 7B). In addition, SH48 treatment resulted in an increased accumulation of the autophagic markers LC3II and p62 as determined by Western blot analysis (Figure 7C).

In contrast to SH48, which bears an α,β-unsaturated carbonyl moiety and hence is capable of directly binding to the STAT3, the non-electrophilic analogues SH42 and SH43, which lack such an electrophilic moiety, failed to suppress STAT3 phosphorylation and upregulate p62 expression. Moreover, the SH48-induced accumulation of p62 was abolished by DTT treatment (Figure 7D). Thus, the α,β-unsaturated carbonyl group of SH48 is likely to be important in terms of its induction of autophagy as well as STAT3 inhibition. In an attempt to determine the association between the inhibition of STAT3 signaling and the induction of autophagy by SH48, the interaction between p62 and STAT3 by immunoprecipitation was examined. As shown in Figure 7E, we found a novel binding between STAT3 and p62 in MCF10A-*ras* cells. SH48 treatment suppressed this interaction in a concentration-dependent manner (Figure 7E).

**Figure 8:**
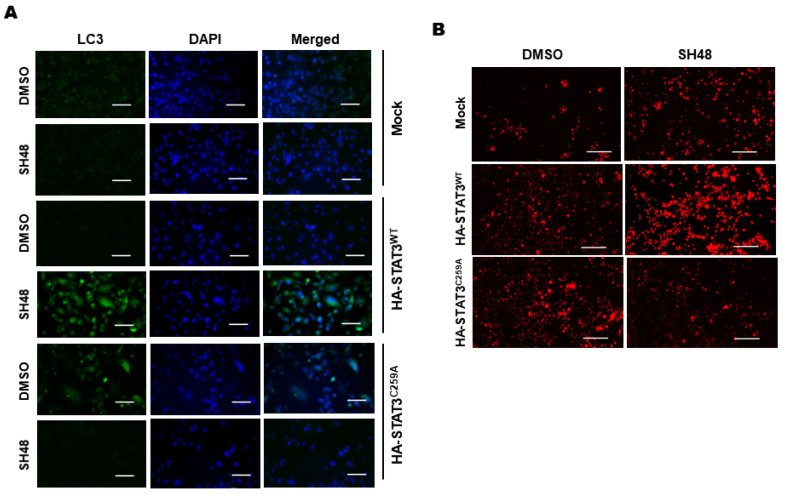
Role of cysteine 259 of STAT3 in SH48-induced autophagy. STAT3-null PC-3 cells were transfected with a mock vector or an expression vector for HA-STAT3^WT^ or mutant STAT3^C259A^ for 18 h. (**A**) The cells were treated with SH48 (10 μM) for 24 h, The transfected cells were treated with DMSO or SH48 for 24 h, and then stained for LC3II (**A**) or lysotracker (**B**). Scale bar, 100 μm.

To investigate the functional importance of Cys259 of STAT3 for SH48-induced autophagy, genetically engineered STAT3 proteins were created and transduced in STAT3-null PC-3 cells. SH48-induced autophagy was verified by the staining of LC3II (Figure 8A) and LysoTracker (Figure 8B). However, these events were not evident in mutant cells, in which Cys259 of STAT3 was replaced to alanine (Figure 8).

## 4. Discussion

Constitutive overactivation of STAT3 is known to be implicated in the pathogenesis of tumor development and progression [22,23,24,25,26,27,28]. Therefore, the inhibition of abnormally elevated STAT3 activity or expression represents a rational therapeutic modality for malignancies, including breast cancer [23,25,26,29,30,31,32,33,46]. The blockage of aberrant STAT3 activation by genetic or pharmacological approaches has been achieved by the use of small molecular weight inhibitors, protein inhibitors, dominant-negative STAT3 mutants, antisense RNA, and interference oligonucleotides (RNAi).

Natural products and their synthetic analogues have been identified as important sources of developing STAT3 inhibitors [47]. For instance, curcumin, betulinic acid, capsaicin, caffeic acid, celastrol, cucurbitacins, guggulsterone, diosgenin, and honokiol are known to be natural STAT3 inhibitors [47]. Deguelin, a rotenoid isolated from several tropical plants of the *Leguminosae* family, was reported to inhibit STAT3 signaling, which accounts for its antiproliferative activity towards some transformed and cancerous cells [4,5,6,7,8,9,10,11,12,13,14,15,16]. In an attempt to enhance its efficacy while minimizing the side effects, a series of analogues of deguelin have been synthesized and tested for their capabilities to suppress STAT3 activation [40]. Among them, an electrophilic analogue, SH48, bearing an α,β-unsaturated carbonyl group was found to be most potent in terms of inhibiting the transcriptional activity of STAT3. However, the mechanisms underlying SH48-induced STAT3 inhibition have not been fully elucidated. Therefore, we intended to conduct an experiment to determine the effects of SH48 on upstream events of STAT3 activation in MCF10A-*ras* cells.

SH48 has a structure that is similar to that of deguelin as it bears an electrophilic α,β unsaturated carbonyl moiety. Unlike deguelin, however, the double bond conjugated to the carbonyl group of SH48 is not incorporated into the aromatic ring but present in a truncated B,C ring (Figure 1). The phosphorylation of STAT3 at the tyrosine705 residue is known to be a crucial event for its activation. As SH48 does not greatly influence the activation of upstream kinases, such as Jaus kinase 2 (JAK2), involved in STAT3 phosphorylation, we speculated that the compound SH48 could form a complex with STAT3, presumably at a critical thiol residue, thereby suppressing the phosphorylation and subsequent dimerization of STAT3. The STAT3 dimer is formed via the reciprocal interaction between the SH2 domain of one monomer and the phosphorylated tyrosine of the other. We speculate that the interaction of SH48 with cysteine 259 of STAT3 hampers the access of an upstream kinase to Y705 for phosphorylation.

It is well known that STAT3 is involved in the transcriptional regulation of genes encoding apoptosis inhibitors (e.g., Bcl-xL) and cell-cycle regulators (e.g., Cyclin D1) [24,25,26]. However, the SH48-induced suppression of STAT3 did not lead to apoptosis but rather induced autophagy in MCF10A-*ras* cells. The induction of autophagic cell death by SH48 was verified by the upregulation of LC3II and p62, which are the autophagy markers. LC3II and p62 constitute autophagosome, which is an important factor in autophagic signaling.

As autophagy progresses, autophagosome is fused with lysosome and degraded. Thus, the accumulation of p62 is anticipated to be reduced as autophagy terminates [48,49]. In line with this speculation, the expression level of p62 was shown to increase until 24 h and gradually decrease by 36 h (S.-J. Hong, unpublished observation). When autophagosome is degraded, LC3II maintains a constant level because LC3II is degraded to a lesser extent than p62 during the destruction of autophagosome.

SH48 has an α,β-unsaturated carbonyl moiety which can mediate the process of Michael addition between SH48 and STAT3. Although our study suggests that SH48 inhibits STAT3 by Michael addition, hydrogen bonding may also be involved. This is a weak force but has enough energy to induce a reaction between molecules. Therefore, the cell viability was compared after treatment with an equimolar concentration of SH48 and SSI1205, which are incapable of interacting with STAT3 via H-bonding. However, the H-bond is unlikely to contribute to the inactivation of STAT3.

**Figure 9:**
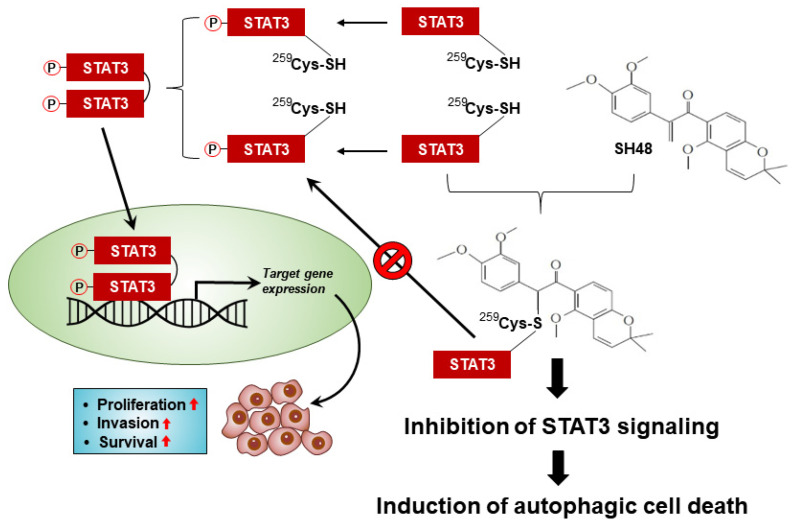
Proposed scheme for SH48-induced autophagy through the inhibition of STAT3 activation. Phosphorylation of STAT3 on Tyr705 facilitates its homodimerization, nuclear translocation and transcriptional activity. STAT3 modified by SH48—presumably at Cys259—cannot undergo the phosphorylation and transcriptional activation required for cancer cell proliferation, invasion, and survival. This results in autophagic cell death.

In summary, the synthetic deguelin analogue SH48 inhibits the phosphorylation, DNA-binding activity, and transcriptional activity of STAT3, leading to the induction of autophagy in MCF10A-*ras* cells (Figure 9). SH48 induces autophagy in MCF10A-*ras* cells but not in MCF10A normal cells. Structure–activity analysis revealed that the α,β-unsaturated carbonyl moiety of SH48 is essential for its complex formation with STAT3 and consequent inactivation of this oncogenic transcription factor. Further studies will be necessary to clarify the direct interaction of SH48 with STAT3 by using more sophisticated physicochemical techniques.
